# The role of alliance management, big data analytics and information visibility on new-product development capability

**DOI:** 10.1007/s10479-021-04390-9

**Published:** 2021-11-19

**Authors:** Rameshwar Dubey, David J. Bryde, Gary Graham, Cyril Foropon, Sushma Kumari, Omprakash Gupta

**Affiliations:** 1 Liverpool Business School, Liverpool John Moore’s University, Liverpool, Merseyside L3 5UG UK; 2 Leeds University Business School, University of Leeds, Maurice Keyworth Building, Leeds, LS2 9JT UK; 3 Montpellier Business School, Montpellier Research in Management, 2300 Avenue des Moulins, 34185 Montpellier, France; 4 Faculty of Business, Law and Politics, University of Hull, Hull, HU6 7RX UK; 5 College of Business, University of Houston-Downtown, Houston, USA

**Keywords:** NPD, Dynamic capability view, Alliance management capability, Big data analytics capability, Information visibility, PLS-SEM

## Abstract

Many organizations are increasingly investing in building dynamic capabilities to gain competitive advantage. New products play an important role in gaining competitive advantage and can significantly boost organizational performance. Although new product development (NPD) is widely recognized as a potentially vital source of competitive advantage, organizations face challenges in terms of developing the right antecedents or capabilities to influence NPD performance. Our research suggests that organizations should invest in building alliance management capability (AMC), big data analytics capability (BDAC) and information visibility (IV) to achieve their desired NPD success. Informed by the dynamic capabilities view of the firm (DCV) we have stated seven research hypotheses. We further tested our hypotheses using 219 usable respondents gathered using a pre-tested instrument. The hypotheses were tested using variance based structural equation modelling (PLS-SEM). The results of our study paint an interesting picture. Our study makes some significant contribution to the DCV and offers some useful directions to practitioners engaged in NPD in the big data analytics era. We demonstrate that AMC and BDAC are lower-order dynamic capabilities and that AMC has a positive and significant influence on BDAC. In turn, AMC and BDAC influence NPD under the moderating influence of IV. Ours is one of the first studies to empirically establish an association among three distinct dynamic capabilities which are often considered in isolation: AMC, BDAC and NPD. Our findings support emergent views on dynamic capabilities and their classification into various orders. Lastly, we provide empirical evidence that information visibility acts as a contingent variable to both AMC and BDAC effects on NPD. We end our paper by outlining some limitations of our study and by offering useful future research directions.

## Introduction

In recent years, new products are being rapidly introduced into markets, as many business enterprises strive to achieve a significant growth in their market share through new product development (NPD) (Bhuiyan, 2011; Zhao & Chadwick, 2014). Managing NPD is often touted as one of the major sources of competitive advantage (Thomke, 1998), with experimentation, learning, and prototyping being integral components of the development and innovation of *products* and *services*. Schilke (2014) argues that NPD can be a key organizational capability and lead to competitive advantage. For instance, through introducing new ideas to the market quickly, with a focus on high customer satisfaction, and on developing products which are easier to manufacture, use and repair than existing ones (Thomke, 2007).

Managing NPD is a complex task, typically requiring huge investment and the return on investment (ROI) is highly uncertain. The harsh realities are that the majority of new products never make it to market and those that do face a failure rate somewhere in the order of 25 to 45 percent (Cooper, 2001). For every seven new product ideas, about four enter development, one and a half are launched, and only one succeeds (Booz et al., 1982; Urbig et al., 2013). Despite the extensive research on how to achieve success in NPD, firms continue to deliver products that fail and therefore NPD ranks among the riskiest and most complex tasks for many companies.

As the number of dollars invested in NPD goes up, the pressure to maximize the return on those investments also goes up. The pressure becomes heightened as an estimated 46 percent of resources allocated to NPD are spent on products that are cancelled or fail to yield an adequate financial return. The extant literature on NPD and its contribution to the growth of companies, its influence on profit, and its role as a key factor in business planning have been well-documented (Bhuiyan, 2011; Cooper, 2001; Crawford, 1992; Ulrich & Eppinger, 2011; Urban & Hauser, 1993). Bhuiyan (2011) argues that new products are responsible for employment, economic growth, technological progress, and high standards of living. Therefore, the study of NPD and the processes through which new products emerge is important.

The adoption of information and communication technologies (ICTs) can significantly boost innovation productivity and performance (Ollo-Lopez and Aramendia-Muneta, 2012). At firm level the use of ICTs helps to exploit the resources and capabilities of the firm to gain a competitive advantage (Barney et al. 2001). The existing literature review indicates a positive link between the effective use of ICTs and performance at the firm and macro levels. In recent years the use of radio frequency identification (RFID) tags, cloud computing, the internet of things (IoT), and the use of big data & business analytics has emerged as one of the most important platforms for innovative services (Akter et al., 2020). We acknowledge the importance of visibility as either a limiting or enabling factor associated with the quality of and ultimate influence on big data analytics capability (Srinivasan and Swink, 2018). Moreover, visibility also plays a significant role on the degree of the collaboration among the partners engaged for NPDs (Caridi et al., 2017).

Acur et al. (2010) argues that organizations can leverage their technological competence to positively influence their NPD programs. Despite making an increasing contribution to the field, scholars have not paid enough attention to the question of how IT or emerging technology can be leveraged to build NPD capability (Aljumah et al., 2021; Wetzels, 2021). Overall, our critical review of literature suggests that the role of alliances among partners engaged in developing the NPD capability has not received significant attention. Kalaignanam et al. (2007) provides support to the argument that inter-organizational alliances are critical to the NPD. Rothaermal and Deeds (2006) argue that alliance management capability has mixed results on the development of NPD capability. However, given that a lot of time has elapsed since that study, we suggest the need for contemporary work that evaluates the role of alliances in building NPD capability. In this study, therefore, we aim to investigate how the alliances between large, well-established firms and small firms influence their joint NPD capability under the influence of visibility. Following on from scholarly debates (see, Schilke, 2014; Fainshmidt et al., 2016; Dubey et al., 2021), we argue that alliance management capability (AMC) and big data analytics capability (BDAC) are dynamic characterisitics which lead to NPD. Given its seminal nature, we adopt the Rothaermal and Deeds (2006, p. 430) definition for AMC as a “*firm's ability to effectively manage multiple alliances”.* Kalagnanam et al. (2007) have empirically tested the impact of asymmetric alliances between the large and small firms on the shareholders’ value of the partners firm. However, the literature focusing on AMC in the context of NPD does not provide clear answers to some fundamental queries, related to the role of AMC on building NPD capability. We note this as a clear research gap and to address it we posit our first research question (RQ1) as: *How does AMC influence NPD capability development?*

Pavlou and El Sawy (2006), in one of their studies, have demonstrated the use of IT capability to influence NPD capability. This generated competitive advantage for firms competing in dynamic turbulent environments. NPD in the age of big data has gained significant momentum (see, Johnson et al., 2017; Zhan et al., 2018; Sun & Liu, 2020; Giannakis et al., 2020; Liu et al., 2020; Cappa et al., 2021). Johnson et al. (2017) further argue that organizations are increasingly investing in big data analytics capability to transform their NPD activities. In support of the claims by Johnson et al. (2017), Zhan et al. (2018) further argue that BDAC has been increasingly utilized to understand the latent needs of the customers. Barczak et al. (2008) in their studies have examined the effects of information technology (IT) usage for new product development (NPD) in a global context. However, the study reveals an interesting outcome as the impact of the usage of IT for NPD in different countries was different. The Barczak et al. (2008) findings suggest that those firms use more globally dispersed teams for NPD and outsource more of their development activities, IT usage is likely to increase to facilitate communication and cooperation. Yet, despite the increasing use of BDAC in NPD activities, the existing studies have remained silent on how BDAC can influence NPD capability. Moreover, how AMC capability influences BDAC and subsequently NPD capability is not well understood. We note this as a research gap. To address it we posit our second research question as (RQ2): *How does AMC effect NPD capability under the mediating influence of BDAC?*

Barratt and Oke (2007) argued that how visibility in the supply chain network plays a significant role in gaining competitive advantage. Swanithan and Tayur (2003) argue that visibility can be influenced by the access to information across the design chain and if used effectively this can improve collaboration. This argument is corroborated by Caridi et al. (2017) who state that the extent of information sharing among the partners involved in the NPD is critical for success (Smits & Kok, 2012). Firms often lack trust with their partners and this can inhibit them. Therefore they must share information with their partners in order to gain maximum competitive advantage and prevent undesired outcomes (Li & Qiu, 2006). Pemartin and Rodríguez-Escudero (2021) argue that continuous interactions and information sharing, with a high degree of visibility, reduces the opportunistic behaviour among the partners involved in NPD. Despite the increasing importance of information visibility, the literature focusing on the visibility in the context of NPD is limited (Caridi et al., 2017). We note this as a clear research gap, which leades to our hird research question (RQ3): *How does information visibility among partners influence the level of alliance management capability and their joint NPD capability?*

Our study’s contributions to knowledge are twofold. Firstly, we offer a significant contribution to the dynamic capability view (DCV) of the firm. We provide an integrative view of alliance management capability (AMC), big data analytics capability (BDAC), information visibility (IV) and NPD. Integrating these different views, we suggest that AMC and BDAC, under the moderating influence of IV, can positively influence joint NPD. Secondly, we contribute to the theoretical understanding of the role that alliance management capability and big data analytics has on NPD, which is an under-explored research area in the academic literature. To address our stated research questions we have used 219 responses from auto components manufacturing organisations located in India. To theoretically substantiate our results we have grounded our theoretical model in the DCV of the firm (Teece et al., 1997).

Our paper is organised as follows. In Sect. 2 we discuss underpinning theories, a theoretical model and we introduce our research hypotheses. In Sect. 3, we present our research design which includes discussion of instrument design, measures, data collection procedures and the demographic profile of the respondents. In Sect. 4, we present our data analysis. In Sect. 5, we discuss our results, including our theoretical contributions, managerial implications, limitations of the study and further research directions.

## Theory development and hypotheses formulation

The focus in our investigation is with alliances and big data capabilities and the reasons for this have been outlined in the previous section. The foundation of our theoretical model (presented in Sect. 2.5) is the DCV of the firm (Teece et al., 1997). DCV has gained significant attention from the operations and supply chain management community (see, Eckstein et al., 2015; Akter et al., 2016; Gu et al., 2021; Dubey et al., 2021), following Teece et al.’s (1997) initial theoretical contribution to strategic management. The DCV is an extension of the classical resource-based view (RBV) of the firm (see, Barney, 1991). Helfat and Peteraf (2003) argue that the DCV involves *adaptation* and *change*, because it *builds*, *integrates*, *or reconfigures the strategic resources* and *capabilities* needed to build competitive advantage. Teece (1997, p. 516) defined DCV as “*the firm’s ability to integrate, build and reconfigure internal and external competencies to address rapidly changing environments”.*

DCV may be understood as the combination of specific processes or routines that facilitate integration, conversion, or renewal of tangible and intangible resources into new organisational capabilities as the external environment changes (Eisenhardt & Martin, 2000). Hence, in a highly turbulent environment, the dynamic capabilities are *simple*, *experiential*, *unstable processes* that are acquired via quick learning from unexpected situations that produce unexpected results (Eisenhardt & Martin, 2000; Fosso Wamba et al., 2020a, 2020b).

The underlying principles of DCV revolve around two basic aspects: (1) the relationship between dynamic capabilities and organisational performance and (2) the dynamic capabilities and their effects being highly visible in technologically intensive industries (see, Fainshmidt et al., 2016; Dubey et al., 2021). Dubey et al., (2021, p. 137) argue that the DCV literature provides an explanation as to: “*how the hierarchical ordering of dynamic capabilities and the economic context serve as contingencies producing differential outcomes*”.

### Alliance Management Capability (AMC)

Management scholars have argued that a firm’s alliance capability is one of the major sources of competitive advantage (Schreiner et al., 2009). To further understand the role of AMC in the context of our study, we build on Schilke’s (2014, pp.183–184) arguments that: “*organizations with a strong alliance management capability possess routines that support various alliance-related tasks, such as partner identification and inter-organizational learning, that facilitate an effective execution of inter-firm relationships*”. Dubey et al. (2021), in one of their recent studies, found that AMC is a higher order dynamic capability which has a significant effect on the adoption of supply chain analytics capability. The firms that significantly invest in the AMC, develop routines that provide adequate support to various activities such as selection of partners and inter-organizational learning which are considered as a vital ingredient of interfirm relationships (Bicen et al., 2021; Schilke, 2014; Zhang et al., 2021). Although, we understand that AMC is a source of competitive advantage for those firms engaged in the NPD in extremely turbulent environments, building and maintaining AMC usually requires significant investments. Owing to the high investments we believe that decision related to investments in AMC requires careful consideration of the external and internal factors. Hence, the environmental turbulence significantly affects the extent the alliance opportunities (Schilke, 2014). Building on the research findings of Dubey et al. (2021) we argue that AMC may have desired positive effects on BDAC. In simple words, we can understand that in turbulent environments, AMC helps resolve any level of conflict among the key stakeholders engaged in NPD. The existing works on AMC provide rich association between AMC and organizational performance (Niesten & Jolink, 2015; Sivadas & Dwyer, 2000). Petersen et al. (2003) argue that the involvement of suppliers in the early stage of NPD can help reduce its cost. Although the integration of the stakeholders in the NPD is a well understood practice, the number of empirical studies focusing on the influence of alliance capability on NPD performance is limited (Rothaermel & Deeds, 2006). Alliance management may occur over one or more activities within industry actors, including NPD, asset management and risk sharing. Despite the potential benefits from undertaking AMC there exists many forms of challenges, resulting from the poor coordination of the AMC activites (Dubey et al., 2021).

Therefore following these preceding arguments we hypothesize the following:

#### Hypotheses 1

AMC has a positive and significant impact on NPD

#### Hypotheses 2

AMC has a positive and significant impact on BDAC

### Big Data Analytics Capability (BDAC)

In recent years, information technology has advanced rapidly and made significant progress in improving coordination between industry partners. However, information technology alone cannot provide competitive advantage to a firm. To gain competitive advantage in this “age of digital revolution”, big data analytics capability is considered to be the game changer; due to its enormous capability to process large complex datasets from which operations managers can draw useful information (Fosso Wamba et al., 2020a, 2020b).

Firms are collecting masses of data from the internet, smart phones, cloud computing and the Internet of Things (IoT). Gupta and George (2016) argues that firm’s need to focus attention of both soft and hard enablers in order to build analytics capability. To develop such a capability, a firm firstly needs to recognize the strategic significance of big data resources for competitive advantage, develop competencies in big data technologies, acquire knowledge on tapping value from big data and transform itself towards a data-driven culture (Aljumah et al., 2021; Dremel et al., 2017; Jeble et al., 2018).

The information derived via processing large complex data sets often helps managers to make key decisions in a highly uncertain and turbulent environment (Fosso Wamba et al., 2018). According to Akter and Wamba (2016), firms need to focus on cutting edge technology, quality analytics resources and analytics-driven management culture for developing big data analytics capabilities. Akter et al. (2016) argued in one of their studies that BDAC is a dynamic capability which helps firms to achieve competitive advantage. On the other hand, Mikalef et al. (2020) argues there is a need to understand how firms may create dynamic capabilities using big data analytics. Therefore, there are two different definitional views of the role of BDAC. We combine these views using a hierarchical perspective of DCV (Dubey et al., 2021; Fainshmidt et al., 2016). In recent studies scholars clearly advocate the positive role and influence of big data analytics capability on NPD (Johnson et al., 2017; Zhan et al., 2018). Hence, we hypothesize this as follows:

#### Hypotheses 3

BDAC has a positive and significant impact on NPD.

### Information Visibility (IV)

IV is a crucial element of partner collaboration (Lee et al., 1997; Wang & Wei, 2007) and can further enhance business performance (Dubey et al., 2020; Straub et al., 2002; Wang & Wei, 2007). Wang and Wei (2007, p. 648) define IV as: “*where an information demander in a supply chain has accurate up-to-date information of all critical activities and processes, such as purchasing, manufacturing, and distribution*”. Barratt and Barratt (2011) argue that visibility is developed via external relations, which include all connected information systems, overlapping planning processes and coordinated decision making.

Managers involved in NPD often seek to improve their visibility in terms of the availability and quality (accuracy, usefulness) of information (Caridi et al., 2014, 2017). The organizations that invest in information visibility are well positioned to develop and deploy systems and processes that support their analytics capability (Srinivasan and Swink, 2018) and further support alliance formation in the NPD (Caridi et al., 2017; Peng et al., 2014). Hence, we can argue that IV further enhances the AMC and BDAC capabilities that support NPD activities. We hypothesize these preceding discussions as follows:

#### Hypotheses 4

The function and role of IV positively moderates the association between AMC and NPD;

#### Hypotheses 5

The function and role of IV positively moderates the association between BDAC and NPD;

### New Product Development (NPD)

It has long been argued that NPD is one of the most important organizational activities that helps the organization to gain competitive advantage (Lawson & Samson, 2001). NPD includes range of processes in order to firstly, bring a new product to market or secondly, in re-inventing the existing product to suit current market needs or thirdly, to introduce a product completely to a new markets (Durmusoglu, 2009).

Durmusoglu (2009, p. 366), defined NPD as follows: “*NPD is a long process consisting of various activities such as product line planning, strategy development, concept generation and screening, business analysis, development, testing, validation, manufacturing development, and commercialization*”. Schilke (2014) argues that NPD requires sincere efforts in terms of long-term commitment from within the organization. For instance, resources like skilled personnel, specialized facilities, infrastructure etc. are vitally important for the NPD success (Helfat et al. 2007). It is well understood that each NPD is different in terms of the information, as well as the cooporation, needed among the various partners engaged in NPD (Durmusoglu, 2009; Schilke, 2014; Johnson et al., 2017). In recent times, the focus on attaining faster NPD cycle times is driven by reduced product life cycles (Griffin & Page, 1993). Numerous studies have established that the fast development of new products leads to competitive advantage (Henard & Szymanski, 2001). Based on previous studies, we hypothesize that NPD has a positive and significant effect on market performance and financial performance as follows:

#### Hypotheses 6

NPD has a positive and significant effect on the market performance;

#### Hypotheses 7

NPD has a positive and significant effect on the financial performance.

## Research design

We tested our research hypotheses using cross-sectional data gathered using a survey-based instrument. We developed the instrument using measures for the variables following an extensive review of the literature. The process of instrument development took place in three stages. Firstly, we conducted field interviews with 17 senior managers from automotive components manufacturing companies. Each interview was between 30 and 45 min and was split into two parts. Firstly, we asked the managers to describe the routine activities that are key for their organizations to adapt to the rapid changing business environment. Interestingly, most of these managers have suggested three key activities. These three activities are named as NPD, technological innovation and alliance management. NPD and technological innovation were among the most frequently cited responses. Secondly, we asked these managers to fill out the initial draft of the questionnaire to be used for the final survey. The overall objective of this preliminary exercise was to assess to what extent the wordings in the questionnaire were understandable. As a result of this exercise, some of the items were reworded, though no items were removed. The list of items is provided in Appendix A.

### Measures

We used multi-item constructs to test our research hypotheses. The measures were adapted from existing studies. We triangulated the inputs obtained from the managers with complementary data sources (Dubey et al., 2021; Schilke, 2014) to assure that the measures are reliable. Next, we discuss our measures for each model variable.

#### Alliance Management Capability (AMC)

We adopted a five-items reflective construct to measure AMC, as developed by Schilke and Goerzen (2010). The measuring items were as follows: (*a) coordination; (b) alliance portfolio coordination; (c) corganizational learning; (d) alliance pro-activeness; and (e) alliance transformation*.

#### Big Data Analytics Capability (BDAC)

For BDAC we modified the measures developed by Gupta and George (2016). This is a five items reflective construct. We included these items to understand how: (a) *tangible resources*; (b) *human skills*; (c) *technical skills*; (d) *data-driven culture* and (e) *organizational learning* can help develop BDAC.

#### New Product Development (NPD)

To measure NPD we adopted the four items construct measurement developed by He and Wong (2004). These items are: (a) *introduction of new products*; (b) *expanding product range*; (c*) entering into new markets* and (d) *entering new technology fields*.

#### Information Visibility (IV)

To measure IV, we adopted four items from Wang and Wei (2007) and Srinivasan and Swink (2018), which are relevant to our context. The measuring items are: (a) *new product information*; (b) *product design information*; (c) *market intelligence* and (d) *bill-of-materials (BOM) information*.

#### Competitive Advantage (CA)

We operationalized CA as a two-dimensional construct: (a) market performance and (b) financial performance, both of which are measured in comparison to competition. We have adapted these items from Sarkar et al. (2001), Schilke (2014) and Dubey et al. (2019). The measuring items for market performance are (a) market share; (b) sales growth and (c) market development. Whilst the measuring items for financial performance are: (a) EBIT; (b) ROI; (c) ROS.

#### Control Variables

Consistent with previous studies (see, Schilke, 2014; Dubey et al., 2021) we considered firm size, alliance portfolio size and market scope to be control variables in the model.

##### Firm Size (FS)

Chang and Thomas (1989) argue that the firm size can enhance competitive advantage. For example, larger sized firms have more ability to access resources at lower cost compared to smaller sized firms.

##### Alliance Portfolio Size (APS)

Previous studies have noted a significant relationship between the number of firm’s alliances and their organizational performance (Powell et al., 1996). We therefore controlled for this variable, measuring APS by the current firm’s total number of alliances, adopting Schilke’s (2014) suggestions. In order to reduce the skewness in answers, we converted the results to the logarithmic value.

##### Market Scope (MS)

We controlled the breadth of the firm’s product offerings and target market, as key dimensions that may affect the competitive advantage of the firm (Schilke, 2014). We adapted the measures from Zott and Amit (2008) for the MS construct.

### Sampling design and data collection

Who chose Indian auto-components sector for the study. The autocomponents industry market landscape is dramatically changing coming out of the COVID-19 pandemic crisis. The sector is currently involved in a ramp up and rapid acceleration in NPD, as it is having to respond to a revolution in vehicle manufacturing e.g. the rapid transitioning taking place to connected and autonomous motoring and electric/hybrid vehicles. This is driven by consumer and governmental pressure for change and the resultant migration from internal combustion engines (ICEs) to the battery driven electric vehicles. Also there is the market pressure from the original equipment manufacturers (OEMs) for suppliers’ to compress their product development cycles down from 4 to 2 years for new makes and models. Furthermore, they are requiring more supplier investment in R & D and innovation to remain competitive and meet the institutional pressure to change their product, service offering and improve their sustainability and environmental performance. There is pressure on the sector from multiple stakeholders i.e. governments through to inter-governmental groups, such as the UN (see, Iyer et al., 2013; Dubey et al., 2018, 2021), to reduce emissions, for example. There are institutional pressures for efficiency savings, especially with the cash constraints resulting from demand collapses during the COVID-19 pandemic; and over capacity in the market is contributing to the accelerating IoT roll out and the use of sensor technology in auto components. Pressure is being applied to improve their product and component design for circularity, re-use etc. To summarize, the auto-component sector is experiencing rapid industrial change, at an unprecedented scale and speed, and this is pressuring them to increase their R & D expenditure on NPD in order to remain competitive and survive the crisis they are facing.

Considering the huge population size of firms operating in the autocomponents manufacturing industry, a simple random sampling method was selected for this study. We deployed various selection factors to reach at a sample size fit for our study. Every manufacturing unit which we selected has hundreds of employees, however only a few employees are working in the NPD department. Our study relates to NPD activity in their organization. Due to the interdisciplinary nature of our research, only a select group of senior level managers would have an in-depth knowledge of concepts and terms related to NPD, AMC, BDAC and IV (Johnson et al., 2017; Srinivasan and Swink, 2018; Dubey et al., 2021). Hence, only a relatively small percentage of NPD managers in the auto components manufacturing industry could respond to our survey. Peng and Lai (2012) have noted the challenges in obtaining a large sample size from organizations. We took some special efforts to identify suitable firms and senior level supply chain managers from the ACMA (The Autocomponents Manufacturers Association in India) for the purpose of our study. The Association acted as a gate-keeper, identifying respondents with the perquisite knowledge and NPD expertise, plus the necessary executive rank, to ensure sample reliability and external response validity.

**Fig. 1:**
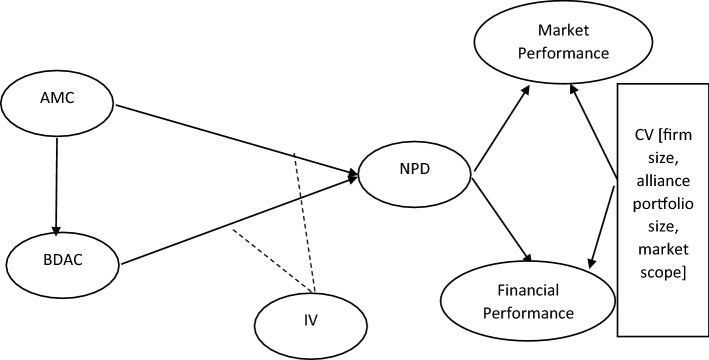
Research Model

Our research model (see Fig. 1) includes a path model with linkages that include multiple relationships, which cannot be easily analyzed using traditional methods, such as multiple regression techniques. Rather, in recent studies of a similar nature to our research, variance based structural equation modelling (PLS-SEM) has been used (Peng and Lai, 2012; Akter et al., 2017; Kock, 2019). Cohen’s (1992) procedure for statistical power analyses recommends a sample size of 147 for a minimum R^2^ of 10% with 5% significance level. Moreover, the sample size used by various scholars in similar studies varies from 100 to 405 respondents.

We distributed our questionnaire among the 654 manufacturing units. We obtained 219 valid responses (33.49%) after two follow-ups. The response rate is consistent with similar survey based studies (Srinivasan and Swink, 2018) and above the minimum number of 147 recommended. The characteristics of the participating firms and the respondents are presented in Table 1. Table shows the breakdown of respondents from firms with more than 5000 employees (19.63%), between 1000 and 4999 employees (23.74%), 500–999 employees (8.68%), 250–499 employees (14.61%), 100–249 employees (15.07%) and less than 100 employees (18.26%). Moreover, our respondents generally consisted of senior representatives from their organizations, holding job titles like Head of R&D, R&D Manager, Chief Operations Manager and Chief Information Manager.

**Table 1:** Sample composition

	Sample ( t = 1) (n = 100)	Sample (t = 2) (n = 119)
*Firm size (employees)*		
≥ 5000	20 (9.13%)	23(10.50%)
1000–4999	23(10.50%)	29(13.24%)
500–999	10(4.57%)	9(4.11%)
250–499	15(6.85%)	17(7.76%)
100–249	17(7.76%)	16(7.31%)
< 100	15(6.85%)	25(11.42%)
*Designation*		
Head of R&D	23(10.50%)	43(19.63%)
R&D Manager	25(11.42%)	24(10.96%)
Chief Operations Manager	23(10.50%)	22(10.05%)
Chief Information Manager	29(13.24%)	30(13.70%)

Next we checked our non-responses in two ways. Firstly, we compared the early and late respondents, following Armstrong and Overton (1977) protocol. The results of the t-tests indicated no significant differences (p > 0.05) across means for each of the constructs between early and late respondents. Secondly, we examined whether the late respondents (organizations) varied in terms of size. We observed no significant differences in either variable (p > 0.05). These findings suggest that the non-response bias does not present a significant problem.

## Data analysis

Peng and Lai (2012, p. 468) argue that: “*PLS is a prediction oriented statistical tool that helps researchers to understand the predictive validity of the exogenous constructs*”. Our study examines the association between AMC, BDAC and NPD. In existing literature, where there is no empirical evidence anticipating a relationship, as is the case with AMC and NPD, the variance based structural equation modelling (PLS-SEM) is most suitable (see, Peng and Lai, 2012; Akter et al., 2017; Rigdon et al., 2017; Hult et al., 2018). We examined the model in two-stages, using Warp PLS 7.0: (stage 1) checking the construct validity and (stage 2) analyzing the structural model (see, Kock, 2019; Dubey et al., 2021) (Table 2).

**Table 2:** Measurement properties (N = 219)

Constructs	Items	λi	Variance	Error	Scale composite reliability (SCR)	Average Variance Extracted (AVE)
CO	AMC1a	0.65	0.42	0.58	0.86	0.61
	AMC1b	0.85	0.72	0.28		
	AMC1c	0.77	0.59	0.41		
	AMC1d	0.83	0.69	0.31		
APC	AMC2a	0.89	0.79	0.21	0.95	0.84
	AMC2b	0.94	0.88	0.12		
	AMC2c	0.91	0.83	0.17		
	AMC2d	0.92	0.85	0.15		
OL	AMC3a	0.74	0.55	0.45	0.92	0.74
	AMC3b	0.87	0.76	0.24		
	AMC3c	0.89	0.79	0.21		
	AMC3d	0.92	0.85	0.15		
AP	AMC4a	0.91	0.83	0.17	0.94	0.80
	AMC4b	0.93	0.86	0.14		
	AMC4c	0.73	0.53	0.47		
	AMC4d	0.98	0.96	0.04		
AT	AMC5a	0.97	0.94	0.06	0.91	0.77
	AMC5b	0.97	0.94	0.06		
	AMC5c	0.66	0.44	0.56		
BDAC	BDAC1	0.77	0.59	0.41	0.94	0.76
	BDAC2	0.77	0.59	0.41		
	BDAC3	0.93	0.86	0.14		
	BDAC4	0.94	0.88	0.12		
	BDAC5	0.92	0.85	0.15		
NPD	NPD1	0.95	0.90	0.10	0.96	0.87
	NPD2	0.93	0.86	0.14		
	NPD3	0.89	0.79	0.21		
	NPD4	0.96	0.92	0.08		
MP	MP1	0.97	0.94	0.06	0.97	0.93
	MP2	0.97	0.94	0.06		
	MP3	0.95	0.90	0.10		
FP	FP1	0.87	0.76	0.24	0.85	0.65
	FP2	0.77	0.59	0.41		
	FP3	0.78	0.61	0.39		
IV	IV1	0.82	0.67	0.33	0.91	0.72
	IV2	0.85	0.72	0.28		
	IV3	0.93	0.86	0.14		
	IV4	0.79	0.62	0.38		

CO, Coordination; APC, Alliance portfolio coordination; OL, Organizational learning; AP, Alliance pro-activeness; AT, Alliance transformation; BDAC, Big data analytics capability; NPD-new product development; MP, Market performance; FP, Financial performance; IV-Information visibility; λi, Factor loadings; SCR, Scale composite reliability and AVE, Average variance extracted

### Measurement properties of constructs

We report scale composite reliability (SCR), average variance extracted (AVE) and the factor loadings (λi) of each measuring items of the constructs in Fig. 1. Following Fornell and Larcker (1981), we observed that λi, SCR and AVE are well above the cut-off values (i.e. λi ≥ 0.5; SCR ≥ 0.7 & AVE ≥ 0.5). This clearly establishes the convergent validity of the constructs used in our study. Next we examined the discriminant validity of the constructs. We observed that the square root of AVE (see the leading diagonal of Table 3) is greater in magnitude than all the correlated values in the same row and column (see, Fornell & Larcker, 1981).

**Table 3:** Dicriminant validity (N = 219)

	CO	APC	OL	AP	AT	BDAC	NPD	MP	FP	IV
CO	*0.78*									
APC	0.61	*0.92*								
OL		0.28	0.50	*0.86*						
AP	−0.02	0.03	0.23	*0.89*						
AT	0.01	0.01	−0.04	−0.06	*0.88*					
BDAC	0.10	0.14	0.09	−0.07	−0.02	*0.87*				
NPD	−0.22	−0.31	−0.36	−0.08	−0.03	0.08	*0.93*			
MP	−0.07	−0.09	−0.15	0.02	0.01	0.20	0.20	*0.96*		
FP	0.18	0.16	0.02	0.03	−0.05	0.17	−0.04	0.04	*0.81*	
IV	0.16	0.13	0.03	0.04	0.12	0.14	0.16	0.14	0.16	*0.85*

CO, Coordination; APC, Alliance portfolio coordination; OL, Organizational learning; AP, Alliance pro-activeness; AT, Alliance transformation; BDAC, Big data analytics capability; NPD-new product development; MP, Market performance; FP, Financial performance and IV-Information visibility

### Common Method Bias (CMB)

Jordan and Troth (2020, p. 4) argues that the, “*common method bias (CMB) basically occurs in survey research when all data (independent variables, dependent variables and mediating and moderating variables) are collected using the same method, potentially resulting in the artificial inflation of relationships*”. CMB is a measurement error which threatens the validity of a conclusion drawn from statistical analysis of data (Podsakoff et al., 2003). CMB is the variance that can be attributed to the method of measurement and not the constructs the measures represent. This can affect the true correlation between dependent and independent variables. Among several methods for statistical controls against CMB, Harman’s single factor test is considered the simplest and most commonly used (Podsakoff et al., 2003). This test can be conducted using factor analysis in SPSS. All factors are loaded into factor analysis while selecting the number of factors as “1”. From the results the % of variance for the first component is observed. If the first component accounts for less than 50% of the variables in the model, then the measuring instrument does not have common method bias issue. Following Podsakoff and Organ (1986) suggestions, we have conducted, a single factor Harman’s test. The results yielded that one factor could explain only 32.62% of the variance.

However, according to Fawcett et al. (2014), controlling for CMB, in addition to statistical control, requires procedural remedies. These need to be designed in accordance with the context of the research (Podsakoff et al., 2003). Whilst the single-factor test is easy to apply, the method has attracted severe criticisms from scholars (Jordan & Troth, 2020; Lindell & Whitney, 2001; Podsakoff & Organ, 1986). The Harman’s test is insensitive, and it is unlikely that a single-factor model will fit the data, particularly when the number of variables used in the study increases (Jordan & Troth, 2020). Hence there is no specific guidelines related to the interpretation of the CMB that provides an acceptable percentage of explained variance of a single-factor model.

Therefore we undertook the second procedure (correlation marker technique), as suggested by Lindell and Whitney (2001). In this context we adopted an unrelated variable to partial out correlations that resulted from the CMB. We further extracted the significant values of the correlations, as suggested by Lindell and Whitney (2001). We note minimal differences between the adjusted and unadjusted correlations.

Based on these statistical results, we conclude that CMB has no serious effects on the results of our study.

Guide and Ketokivi (2015) argue that it is important to run the endogeneity test before we proceed with the hypotheses testing. Kock’s (2019) work found that the nonlinear bivariate causality direction ratio (NLBCDR) is one of the useful tests in case of variance-based SEM for examining the causality of the hypothesized relationship. We observed a NLBCDR of 0.99, which is significantly above the threshold value ≥ 0.7. Hence, we argue that causality is not an issue.

We further provide the values for model fit and quality indices supporting this conclusion (Table 4). Average Path Coefficient (APC); Average R—squared (ARS); and Average Block VIF (AVIF) are the three models fit and quality indices estimated in this study. The values of APC and ARS are found to be significant for the model, as the p values are less than 0.05. Further we determined the goodness of fit (GoF) based on Tenenhaus et al. (2005) suggestions. The value of GoF is found to be 0.46. According to Wetzels et al. (2009), the value of GoF of our model is large (see Table 4).

**Table 4:** Model fit and quality indices (N-219)

Model Fit and Quality Indices	Value from analysis	Acceptable if	References
Average Path Coefficient (APC)	0.32, *p* < .001	*p* < .05	Rosnow and Rosenthal (1991)
Average R—squared (ARS)	0.31, *p* < .001	*P* < .05	
Average block VIF (AVIF)	1.88	≤ 5, ideally ≤3.3	Kock and Hadaya (2018)
Tenenhaus et al. (2005) GoF	0.46	0.36 = large, 0.25 = medium, 0.1 = small	Wetzels et al. (2009)

### Hypotheses Testing

We used Warp PLS 7.0 to test the strength and significance of the relationships articulated in our research hypotheses. The PLS based algorithms does not assume a multinormal distribution, in comparison to the parametric-based techniques for significance tests. We further report the PLS based path co-effecients and p-values for the model in Table 5.

**Table 5:** Structural estimates (N = 219)

Hypothesis	Effect of	Effect on	Β	p-value	Results
H1	AMC	NPD	0.74	< 0.01	supported
H2	AMC	BDAC	0.21	< 0.01	supported
H3	BDAC	NPD	0.80	< 0.01	supported
H4	IV*AMC	NPD	0.66	< 0.01	supported
H5	IV*BDAC	NPD	0.17	< 0.05	supported
H6	NPD	MP	0.37	< 0.01	supported
H7	NPD	FP	0.77	< 0.01	supported
	Control variables				
	FS	MP	0.01	0.45	not-significant
	FS	FP	0.10	0.07	not-significant
	APS	MP	0.08	0.12	not-significant
	APS	FP	0.19	< 0.01	significant
	MS	MP	0.05	0.25	not-significant
	MS	FP	0.07	0.15	not-significant

AMC, Alliance management capability; BDAC, Big data analytics capability; NPD, New product development; MP, Market performance; FP, Financial performance; IV, Information visibility; FS, Firm size; APS, Alliance portfolio size and MS, Market scope

Table 5 provides the results in relation to the hypothesized relationships between the dynamic capabilities as specified in (H1-H3), the moderating effect of IV (H4-H5) and between the dynamic capability and competitive advantage (H6-H7). It also reports the test results in respect of the control variables used in our study.

We found support for H1 (β = 0.74; *p* < 0.01). This result suggests that AMC plays a significant role in the success of NPD, which is consistent with the observations of Sivadas and Dwyer (2000). Addressing H2, we found support for the propostion that AMC is positively connected to BDAC (β = 0.21; *p* < 0.01). These findings are consistent with previous arguments in the literature (see, Rothaermel & Deeds, 2006; Dubey et al., 2021). Likewise there was support for H3 (β = 0.80; *p* < 0.01),

Next we found support for the hypothesised relationship between NPD and competitive advantage: H6 (β = 0.37; *p* < 0.01) and H7 (β = 0.77; *p* < 0.01). We found these results are consistent with previous studies (Lawson & Samson, 2001; Schilke, 2014).

We tested hypotheses H4 and H5 for the interaction effects of IV on the paths joining AMC and NPD together with BDAC and NPD. We found that IV has a positive and significant moderating effect on the paths joining AMC and NPD (β = 0.66; *p* < 0.01) and the paths joining BDAC and NPD (β = 0.17; *p* < 0.05) – hence support for H4 and H5.

We found that control variables FS and MS have no significant effects on MP/FP. However, APS has a significant effect on FP but it does not have any significant effect on MP.

## Discussion

Our study has investigated how the formation of alliances among organizations influences their joint big data analytics capability and NPD performance. The role of strategic alliances in high tech industries has gained increased attention in recent years, yet the existing literature does not provide a clear understanding of the role of alliance management capability and big data analytics capability in NPD. We provide empirical evidence that both the AMC and the BDAC are significantly associated with NPD. In a way we have attempted to extend the Barczak et al. (2008) findings. Barczak et al. (2008) argued the importance of the collaboration in leveraging IT capability for the NPDs. Furthermore, Johnson et al. (2017) argued that how 3 V’s charecteristics of the “Big-data” can be exploited for the NPDs. However, despite acknowledgment of the IT capability, the existing literature has not paid much attention to the joint effects of AMC and BDAC as two dynamic capabilities for the NPDs. Furthermore. existing studies have either focused on the 3Vs-volume, velocity, and variety of data and their influence on the NPD (Johnson et al., 2017) or the role played by social media in NPD (Giannakis et al., 2020; Zhan et al., 2018). Rothaermel and Deeds (2006) found that the alliance type and experience act to moderate the relationship between a high-technology venture's R&D alliances and its performance in NPD. Moreover, under what condition the AMC and BDAC will have better influence on NPD was not well understood. Advocates of a more contingent view of the DCV that the potential benefits of the dynamic capabilities (DCs) depend not only on the organizational routine activities but also on the context in which these capabilities are operating (Schilke, 2014). Stemming from the contingent view of the DCs, we consider the role of information visibility as a means to enhance the combined effects of AMC and BDAC on NPD. In doing so we confirm the arguments of Barratt and Oke (2007), that competitive advantage stems from the ways in which existing technologies are used, rather than from the technologies themselves.

Our study does not address the means for NPD directly. Rather, we suggest that NPD, and its effects on competitive advantage, is positively associated with alliance management and big data analytics capability. With inter-organizational factors being important in achieving the desired for success in NPD.

Next, we outline our specific theoretical contributions in two areas.

### Theoretical contributions

The DCV is considered as an extension of RBV. Whilst RBV addresses the ways an organization exploits existing resources, the DCV primarily focuses on the reconfiguration of these strategic resources to achieve a desired competitive advantage (Eisenhardt & Martin, 2000; Schilke, 2014). The existing literature on DCV generally revolves around two basic tenets: (1) the dynamic capabilities leading to competitive advantage; (2) that the value of the dynamic capabilities is more pronounced in the presence of turbulence.

Here our study builds upon previous studies (see, Schilke, 2014; Fainshmidt et al., 2016) in presenting a hierarchichal view of dynamic capabilities. We present AMC, BDAC and NPD as lower-order and higher-order dynamic capabilities. In this respect our study makes a number of important theoretical contributions to the existing literature. Firstly, we demonstrate that the AMC and the BDAC are lower-order dynamic capabilities. However, AMC also has a positive and significant influence on the BDAC. Moreover, AMC and BDAC in turn influence NPD under the moderating influence of IV. This is one of the first studies to empirically establish an association among three distinct dynamic capabilities, which are often considered in isolation. Hence, our findings support emergent views on dynamic capabilities and their classification into various orders.

Secondly, the study provides empirical evidence that IV acts as a contingent variable to both AMC and BDAC effects on NPD; and that IV can further enhance trust and cooperation among the partner alliances involved in NPD. We also found that information process capability often reduces the risk that new products fail because they do not meet evolving customer needs (Smit & Kok, 2012).

Teams engaged in NPD not only face technological and resource uncertainties but also high levels of environmental uncertainties. NPD teams are therefore often unsure about the nature of the market intelligence that may help them to understand the actual needs of customers and their expectations. Moreover, in highly dynamic environments customers also face a dilemma in terms of expressing their actual needs, which may be be attributed to a lack of desired visibility. It is well understood that NPD teams often fail to predict the behaviour of their team members due to lack of information sharing. In such circumstances NPD projects will often fail. Thus we argue that IV further enhances data processing capability, by providing large data sets which can be utilized to draw upon useful information to help NPD teams.

### Managerial implications

Our study provides useful guidance to those managers engaged in NPD. The information asymmetry and the opportunistic behaviours between the partnering firms in an alliance often act as barriers to effective working. Hence, investment in AMC, BDAC and visibility can help minimize these barriers and maximize their NPD success. Our study suggests that managers should consider investing in alliance management capability to achieve desired successes in NPD. However, alliance management capability requires huge investment. Hence, senior managers often face a dilemma regarding the *what, how and when,* in considering investing in AMC. Our results suggest that investments in building dynamic capabilities (such as AMC and BDAC) are well justified in many environments to achieve desired NPD success. Building dynamic capabilities often require the firm to reconfigure its resource base. Hence, practicing managers need to pay close attention to building and exploiting these dynamic capabilities in a way to gain competitive advantage.

Our study has uncovered an important fact that information system managers must not limit themselves to collecting data from their own organizational information systems. They need to extend the scope of data collection, using external sources such as the supply chain, suppliers, customers and social media. Manufacturing firms in India have progressed very well in deploying information systems e.g. most of them have implemented ERP systems over recent years. Some have integrated these systems with their business partners, such as suppliers and customers. This process can be now extended to include data about movement of components inventory within the manufacturing factory or data from the movement of goods across supply chain routes. For this purpose, it is imperative that manufacturing firms introduce several technologies inside their factories as well as over logistics routes. There are several technology enablers available. RFID tags or sensors can be introduced to monitor movement of inventory inside the factory and across supply chains. This will help manufacturing firms in obtaining real-time data on the status of incoming material, improving visibility and reducing inventory across supply chain. Sensors can be used inside factories to detect changes in a particular property (temperature, pressure, level of liquids, speed of vehicles, humidity etc.) to create alerts that would help to improve NPD.

Finally, our study recognizes how to generate inter-organizational governance value, which is essential for the partners engaged in NPD via alliance formation. Timely, accurate, and relevant information is essential for NPD activities. Our results suggest that IV is the outcome of inter-organizational communication among engaged partners in the NPD. Hence, IV must be distinguished from BDAC, because information sharing can be realized through different means, such as technology-based media, social contacts, and procedural venues (Wang & Wei, 2007). Thus, exchanging information on forecasting, planning, product design, and production scheduling reduces information asymmetry and monitoring costs, thus lowering the incentives of the partners engaged in NPD to act opportunistically in their own best interest and detrimentally to the alliance (Dyer, 1997; Wang & Wei, 2007; Srinivasan and Swink, 2018).

### Limitations and further research directions

Like any other studies of a similar nature we recognise that our study has some limitations. However, these limitations and some research questions that we have not addressed in our study may open the window for future research. We believe, therefore, there are several unanswered and new questions that warrant further theorizing and empirical investigation.

Firstly, our study utilized cross-sectional data gathered from a single-informant using a survey-based instrument. Ketokivi and Schroeder (2004) argue that single-informant data contributes to CMB. Moreover, that causality is hard to be determined using cross-sectional data. Hence, we acknowledge that due to the cross-sectional nature of the data, it is quite hard to determine the variable effects of IV on the paths joining AMC and BDAC on the NPD, as this kind of relationships is often assessed via longitudinal data (see Schilke, 2014; Dubey et al., 2021). We therefore strongly recommend expanding our study using longitudinal data or using survey-based data gathered using a multi-informant questionnaire. This will help minimize any CMB in the data.

Secondly, we have analyzed the effects of AMC and BDAC on NPD. However, we suggest future scholars investigate the effects of other dynamic capabilities on NPD. For instance, the effects of organizational agility and adaptability on NPD may yield interesting outcomes.

Thirdly, we have tested our research hypotheses using data gathered from a single industry. We therefore advise our readers to cautiously interpret our results in context to a particular setting and that our results may not be generalized to all settings. Thus future studies may scrutinize the current findings in other settings, possibly incorporating a greater number of different industries, countries, and/or time periods in order to ensure higher level of variance of AMC and BDAC.

Finally, there is a need for a qualitative investigation – exploring a rich and evolutionary phenomena like alliance formation and big data analytics capabilitiy evolution may be the future scope of such a study. Moreover, international comparisons would be really useful here, to examine, amongst other things, cultural differences and their impacts on NPD.

## Conclusions

In conclusion, the behavior of dynamic capabilities and the effect of IV for NPDs are yet to be fully understood. In this study, we posited three research questions based on the research gaps, guided by the arguments of Sandberg and Alvesson (2011). We believe that our research findings open up new debates. In the past, the majority of NPD development activities primarily relied on small data sets, with limited analytics platforms and restricted implementation capability (Johnson et al., 2017). However, in recent years, large data sets and enhanced information processing capability have changed the way NPD activities can be performed (Zhan et al., 2018). However, despite significant advancements in the field of NPD, the academic literature on NPD in the era of technological revolution remains elusive. We believe that our results help address some of the research questions and further provide new avenues for future research.

## Appendix 1: Operationalisation of Constructs

**Table:**

Constructs	Items	Statement	Source
IC	AMC1a	Our organization maintain strong coordination with our partners engaged in the new product development capability	Schilke (2014, p. 189), Dubey et al. (2021)
	AMC1b	Our organization assure tasks fit well with our partners engaged in the new product development	
	AMC1c	Our organization ensure that our work is well aligned with our partners involved in the new product development	
	AMC1d	We regularly interact with the partners to sort out any issues related to the new product development	
APC	AMC2a	We maintain excellent communication among the partners engaged in the new product development	Schilke (2014, p. 189), Dubey et al. (2021)
	AMC2b	We maintain good rapport with our engaged partner's portfolio during the new product development	
	AMC2c	We have clearly defined the roles of each partner involved in the new product development	
	AMC2d	We identify any replication of our efforts and sort out immediately	
IL	AMC3a	We continuously learn from each other during the new product development	Schilke (2014, p. 189), Dubey et al. (2021)
	AMC3b	We are capable enough to absorb new knowledge during the new product development activity	
	AMC3c	We invest significant efforts in analyzing the information provided by the partners during the new product development	
	AMC3d	We try our best to integrate our understanding with the knowledge acquired from our partners during the new product development activity	
AP	AMC4a	Our organization assure that we do not compete with our partners during the new product development	Schilke (2014, p. 189), Dubey et al., (2021)
	AMC4b	Our organization immediately sort out the differences among the partners to improve the alignment	
	AMC4c	Our organization is proactive enough to sense the market strategies of the competitors and their strategies to manage their partners engaged in the new product development	
	AMC4d	Our organization actively monitor environments to explore possibilities of new partnerships to strengthen the new product development initiatives	
AT	AMC5a	Our organization try to build strong and long terms partnerships to boost our confidence in our efforts towards new product development	Schilke (2014, p. 190), Dubey et al. (2021)
	AMC5b	Our organization discusses with the partners while preparing the agreement	
	AMC5c	Our organization is flexible to make changes in the contract terms and conditions to accommodate any degree of exigencies to minimize the barrier that prevents us from building a strong partnership	
BDAC	BDAC1	Our organization is committed to investing in the tangible resources necessary to build big data analytics capability	Gupta and George (2016)
	BDAC2	Our organization invest in human skills to adapt to the dynamic environment	
	BDAC3	Our organization understand the importance of right technical skills which are necessary for extracting useful information from the complex data sets	
	BDAC44	Our organization believe in the enormous potential of big data and its impact on business activities	
	BDAC5C5	Our organization believe in learning and sharing information	
NPD	NPD1	Our organization is actively involved in the introduction of new products	He and Wong (2004)
	NPD2	Our organization is actively expanding its own product range to meet the growing demands of the market	
	NPD3	Our organization is keen to enter into new markets	
	NPD4	Our organization is keen to enter into new technology fields	
IV	IV1	Our organization share the new product information with our partners engaged in the new product development	Wang and Wei (2007)
	IV2	Our organization share product design information with the production and procurement team	
	IV3	Our organization invest in the market intelligence to understand how our competitors are responding to the rapid changes in the business environment	
	IV4	Our organization share thebill-of-materials (BOM) details with the procurement team	
MP	MP1	Market shares	Sarkar et al. (2001)
	MP2	Salet growth	
	MP3	Market development	
FP	FP1	EBIT (Earnings Before Interest and Taxation)	Sarkar et al. (2001)
	FP2	ROI (Return on Investment)	
	FP3	ROS (Return on Sales)	
